# CRTAC1 (Cartilage acidic protein 1) inhibits cell proliferation, migration, invasion and epithelial-mesenchymal transition (EMT) process in bladder cancer by downregulating Yin Yang 1 (YY1) to inactivate the TGF-β pathway

**DOI:** 10.1080/21655979.2021.1974645

**Published:** 2021-11-24

**Authors:** Jianghua Yang, Li Fan, Xiaoxing Liao, Gongjing Cui, Hailong Hu

**Affiliations:** a Tianjin Key Laboratory of Urology, Department of Urology, The Second Hospital of Tianjin Medical University, Tianjin 300211, China; b Department of Urology, Beijing Aerospace General Hospital, Beijing, China; c Department of Urology, Beijing University of Chinese Medicine Third Affiliated Hospital, Beijing, China; d Department of Urology, The Second People’s Hospital of Lianyungang, Lianyungang 222006, Jiangsu, China

**Keywords:** CRTAC1, YY1, bladder cancer, TGF-β pathway

## Abstract

Cartilage acidic protein 1 (CRTAC1) is predicted to be aberrantly expressed in bladder cancer based on bioinformatics analysis. However, its functions and molecular mechanism in bladder cancer remain elusive. This study aimed to explore the role of CRTAC1 in bladder cancer. The mRNA and protein levels of CRTAC1 and Yin Yang 1 (YY1) were detected by reverse transcription quantitative polymerase chain reaction and western blotting. We found that CRTAC1 was downregulated in bladder cancer tissues and cells. Cell Counting Kit-8 assays, colony formation assays, wound healing assays and Transwell assays and western blotting revealed that CRTAC1 overexpression inhibited cell viability, proliferation, migration, invasion and epithelial-mesenchymal transition (EMT) process in bladder cancer, while CRTAC1 knockdown exerted opposite effects on these malignant behaviors. Mechanistically, CRTAC1 targeted YY1 in bladder cancer cells. YY1 was upregulated in bladder cancer tissues and cells. CRTAC1 negatively modulated the mRNA and protein expression of YY1 in bladder cancer cells. Co-localization of CRTAC1 and YY1 expression was assessed using immunofluorescence staining and Co-Immunoprecipitation assays. The interaction between CRTAC1 and YY1 was explored by Chromatin immunoprecipitation and luciferase reporter assays. Moreover, CRTAC1 inactivated the TGF-β pathway by downregulating YY1 expression. Protein levels of factors associated with the TGF-β pathway were examined by western blotting. Rescue assays indicated that CRTAC1 inhibited malignant behaviors of bladder cancer cells by targeting YY1. Overall, CRTAC1 inhibited malignant phenotypes of bladder cancer cells by targeting YY1 to inactivate the TGF-β pathway.

## Introduction

Bladder cancer is a heterogeneous disease that can be classified into non-muscle invasive bladder cancer (NMIBC) and muscle invasive bladder cancer (MIBC) [1]. As the most common urothelial tumor, bladder cancer leads to approximately 200,000 deaths globally in 2018. The incidence and mortality rate in males are four times than those of females [2]. Risk factors such as smoking, family history, occupational exposure, long-term use of urinary catheter all contribute to bladder cancer [3–7]. The clinical outcomes of bladder cancer treatment are still unsatisfactory with less than 10% in 5-year survival in MIBC [8]. Therefore, it is imperative to explore therapeutic targets and investigate the underlying mechanism in bladder cancer.

Cartilage acidic protein 1 (CRTAC1), an extracellular matrix protein of human chondrogenic tissue, has been reported to play an important role in various human diseases. For example, CRTAC1 promotes cell proliferation, migration and extracellular matrix regeneration and remodeling in primary human fibroblasts [9]. CRTAC1 overexpression promotes the pyroptosis of human lens epithelial cells by facilitating the reactive oxygen species (ROS) production in the formation of cataract [10]. High expression of CRTAC1 in patients diagnosed with glioma are associated with longer survival [11]. Based on bioinformatics analyses, CRTAC1 expression is decreased in tissue samples of bladder urothelial carcinoma and its low expression correlates with poor survival in patients with bladder cancer. However, the functions and underlying mechanism of CRTAC1 in bladder cancer have been rarely explored.

Transcription factor Yin Yang 1 (YY1), a member of transcriptional regulatory proteins from the GLI-Kuppel family, contributes to the progression of various biological processes such as cell proliferation, cell migration, cell cycle progress, and cell differentiation [12]. Increasing studies suggest that YY1 functions as a tumor suppressor or a promoter in various cancer progression. For example, YY1 overexpression inhibits cell growth, foci formation and tumorigenesis *in vivo* in breast cancer [13]. YY1 facilitates cell proliferation and migration and suppress cell apoptosis by binding to MYC target 1 in laryngeal cancer [14]. Silenced YY1 promotes cell migration and invasion in pancreatic ductal adenocarcinoma by binding to the promoter region of Feline sarcoma-related (FER) and regulating signal transducer and activator of transcription 3 (STAT3)/matrix metallopeptidase 2 (MMP2) signaling pathway [15]. Additionally, YY1 has been documented to be overexpressed in bladder cancer tissues and high-grade cells, and its expression is associated with disease-free survival in bladder cancer patients. Low YY1 expression is correlated with better prognosis. YY1 regulates epithelial-mesenchymal transition (EMT) markers in bladder cancer by activating the TGFβ pathway [16].

The study aimed to investigate the role of CRTAC1 in the development of bladder cancer. We hypothesized that CRTAC1 might affect malignant behaviors of bladder cancer cells by regulating the expression of downstream target genes. The study may expand our knowledge of the function and underlying mechanism of CRTAC1 in bladder cancer.

## Materials and methods

### Sample collection

Twenty pairs of bladder cancer tissues and adjacent normal tissues were collected from patients with bladder cancer during operation at The Second Hospital of Tianjin Medical University (Tianjin, China). The collected tissue samples were immediately frozen in liquid nitrogen at −80°C for the following assays. This study had been approved by the Ethics Committee of The Second Hospital of Tianjin Medical University (Tianjin, China) and were conducted following code of ethics received from the Ethics Committee. All patients had signed the informed consents before the study.

## Cell culture

Normal urothelial cell line SVHUC-1 (catalog number: CL-0222) and bladder cancer cell lines (T24, catalog number: CL-0227; 5637, catalog number: CL-0002) were purchased from the Procell Co., Ltd (Wuhan, Hubei, China). The cells were incubated in Dulbecco’s modified Eagle’s medium (DMEM, Sigma-Aldrich, Shanghai, China) with 10% fetal bovine serum (Sigma-Aldrich) and 1/100 penicillin/streptomycin (Biochrom, Cambridge, UK) at 37°C in 5% CO_2_.

## Cell transfection

To overexpress CRTAC1 or YY1, the full length of CRTAC1 or YY1 was inserted into pcDNA3.1 vectors to construct pcDNA3.1/CRTAC1 or pcDNA3.1/YY1, and empty pcDNA3.1 vector was set as a negative control (NC). The short hairpin RNAs targeting CRTAC1 (sh-CRTAC1#1/2) were designed and synthesized to silence CRTAC1 expression with sh-NC as a negative control. All above plasmids and vectors have been synthesized by Sangon Biotechnology Co. Ltd. (Shanghai, China). The indicated plasmids and vectors were transfected into T24 and 5637 cells for 48 h using Lipofectamine 2000 (Invitrogen, Carlsbad, CA, USA) according to the manufacturer’s recommendations [17]. The concentration of pcDNA3.1 vectors was 10 nM and that for shRNAs was 40 nM. The transfection efficiency was examined by RT-qPCR after 48 h.

## Reverse transcription quantitative polymerase chain reaction (RT-qPCR)

TRIzol Reagent was used to extract the total RNA from tissues and cells. The reverse transcription of collected RNA was performed with a RETRO-script ^TM^ Reverse Transcription Kit (Invitrogen). PCR was then performed using a PrimeScript Reverse Transcriptase Kit (Takara). PCR was conducted at 95°C for 2 min, followed by 30 cycles of 95°C for 30 s, 30 s at 56°C and 72°C for 1 min [18]. The expression of CRTAC1 and YY1 was calculated with the 2^−∆∆Ct^ method and were normalized to GAPDH [19]. This assay was repeated three times. The sequence of primers used for RT-qPCR are as follows:

CRTAC1:

Forward: 5ʹ-AACTCAGTGCTGGAGATCC-3ʹ,

Reverse: 5ʹ-AGAATCCTTGGCCACACTC-3ʹ;

YY1:

Forward: 5ʹ-AAAGAAACTTCCTCCTGGAG-3ʹ,

Reverse: 5ʹ-GGCTTCATTCTAGCAAATTCTG-3ʹ;

GAPDH:

Forward: 5ʹ-CCTCCTGTTCGACAGTCAG-3ʹ,

Reverse: 5ʹ-CATACGACTGCAAAGACCC-3ʹ;

## Western blotting

Western blotting was performed according to the previous study [20]. RIPA lysis buffer was used to collect total protein from bladder cancer cells and SVHUC-1 cells. The protein concentration was determined by a BCA assay kit (Thermo Fisher Scientific, Shanghai, China). Subsequently, the protein was isolated by 10% SDS-PAGE and then transferred onto polyvinylidene fluoride membranes (Millipore, Billerica, MA, USA). Then, 5% nonfat milk was used to block the membranes for 1 h. The membranes were then cultured with primary antibodies (Abcam, Cambridge, MA, USA), including anti-CRTAC1 (#ab254691, 0.04–0.4 µg/ml), anti-YY1 (#ab109237, 1/2000), anti-E-cadherin (#ab1416, 1/50), anti-N-cadherin (#ab76011, 1/5000), anti-Slug (#ab27568, 1/500), anti-Twist (#ab175430, 1/500), anti-TGF-β (#ab215715, 1/1000), anti-p-SMAD2/3 (#ab254407, 1/1000), anti-SMAD2/3 (#ab202445, 1/1000), anti-p-SMAD1 (#ab226821, 1/1000), anti-SMAD1 (#ab33902, 1/1000) and anti-GAPDH (#ab8245, 1:500) overnight at 4°C. GAPDH served as an internal control. Next, the membranes were cultured with the secondary antibody for 1 h in the dark. The enhanced chemiluminescence solution (Thermo Fisher) was used to visualize the protein bands and ImageJ software (National Institutes of Health, Bethesda, MA, USA) was used for analysis.

## Cell counting kit-8 (CCK-8) assay

The viability of T24 and 5637 cells after indicated transfection was detected by CCK-8 assays. T24 and 5637 cells at the density of 1000 cells/well were grown into 96-well plates and then cultured for indicated time (24, 48 and 72 h). Next, each well was supplemented with 10 μL of cell counting kit-8 solution (Boster, Wuhan, China) and incubated for another 4 h. A microplate reader (Olympus, Tokyo, Japan) was used to assess the absorbance at the wavelength of 450 nm. The CCK-8 assays were repeated at least three times based on the previous study [21].

## Colony formation assay

The transfected T24 and 5637 cells were grown into 6-well plates for 2 weeks. Subsequently, 4% paraformaldehyde was used to fix the colonies for 30 min and then crystal violet solution was used to stain the colonies for 5 min. A microscope (Olympus, Tokyo, Japan) was used to image the visible colonies and the number of colonies was counted manually. This assay was performed at least three times according to the description in previous report [22].

## Immunofluorescent staining

Immunofluorescent staining was used to examine the expression of CRTAC1 and YY1 according to the description in previous studies [23,24]. The T24 and 5637 cells after indicated transfection were fixed with 4% formaldehyde for 15 min, and then permeabilized using 0.5% Triton X-100 for 10 min. Then, cells were blocked with 10% normal goat serum for 30 min. Next, a primary antibody of anti-CRTAC1 (ab254691, 1:40; Abcam) and anti-YY1 (ab109237, 1:100; Abcam) was used to incubate with cells at 4°C overnight. Afterward, cells were rinsed with PBS three times (5 min each) and incubated with the fluorescent-labeled secondary antibody at darkness for 1 h. Finally, DAPI was used to counterstain the nuclei and the images of Immunofluorescent staining were captured by the fluorescence microscope (Leica, Wetzlar, Germany).

## Co-immunoprecipitation (Co-IP)

Co-IP was conducted in reference to the previous study [25]. Total protein was isolated from T24 and 5637 cells, and cell lysates (300 μL) were incubated with 2 μg of YY1 or IgG antibodies for 30 min at 4°C. Next, proteins were incubated with the protein A/G beads (Santa Cruz Biotechnology, Santa Cruz, CA, USA) at 4°C overnight. After washing the beads three times using PBS, the samples were analyzed with Western blotting.

## Chromatin immunoprecipitation (ChIP)

A Magna ChIP Chromatin Immunoprecipitation Kit (Millipore) was used for ChIP analysis according to the previous study [26]. The chromatin was cross-linked with 1% formaldehyde for 15 min at room temperature, and then sonicated into small fragments. Next, anti-IgG or anti-CRTAC1 antibodies were added to incubate with the lysates overnight at 4°C and bound to protein G Sepharose (Invitrogen) at 4°C for 2 h. After decrosslinking, the enrichment of specific fragments was assessed using RT-qPCR.

## Luciferase reporter assay

The binding between CRTAC1 and YY1 promoter was investigated using luciferase reporter assays in reference to previous study [26]. YY1 promoter sequences were inserted into PGL3 vector (Promega, Madison, WI, USA). Then, the vectors carrying YY1 promoter were co-transfected into T24 and 5637 cells with sh-CRTAC1#1/2 or sh-NC using Lipofectamine 2000. Forty-eight h later, the relative luciferase activity of YY1 promoter was detected using the Dual-Luciferase Reporter Assay System (Promega) in T24 and 5637 cells.

## Statistical analysis

Results from at least three independent experiments were presented as the mean ± standard deviation (SD). Statistical analysis was performed using SPSS software, 24.0 (SPSS Inc., Chicago, IL, USA) with one-way analysis of variance (ANOVA) for difference evaluation among multiple groups and Student’s *t*-test for difference evaluation between two groups. Shapiro–Wilk test was used to check the normality of the data. The expression correlation between CRTAC1 and YY1 in bladder cancer tissues was identified by Pearson correlation analysis. *p* < 0.05 was considered as statistically significant.

## Results

The study explored the functions and underlying mechanism of CRTAC1 in bladder cancer. We hypothesized that CRTAC1 might affect malignant phenotypes of bladder cancer cells by regulating its downstream target gene. Our work revealed that CRTAC1 is downregulated in bladder cancer tissues and cells, and CRTAC1 inhibits cell proliferation, migration, invasion and EMT process as shown by functional experiments. Mechanistically, CRTAC1 directly targets YY1 in bladder cancer cells. In addition, CRTAC1 inactivates TGF-β signaling by downregulating YY1. Moreover, rescue experiments implied that CRTAC1 inhibits malignant behaviors of bladder cancer cells by downregulating YY1. In summary, CRTAC1 inhibits cell proliferation, migration, invasion and EMT process in bladder cancer by downregulating YY1 to inactivate the TGF-β pathway.

## CRTAC1 is downregulated in bladder cancer tissues and cells

**Figure 1.**
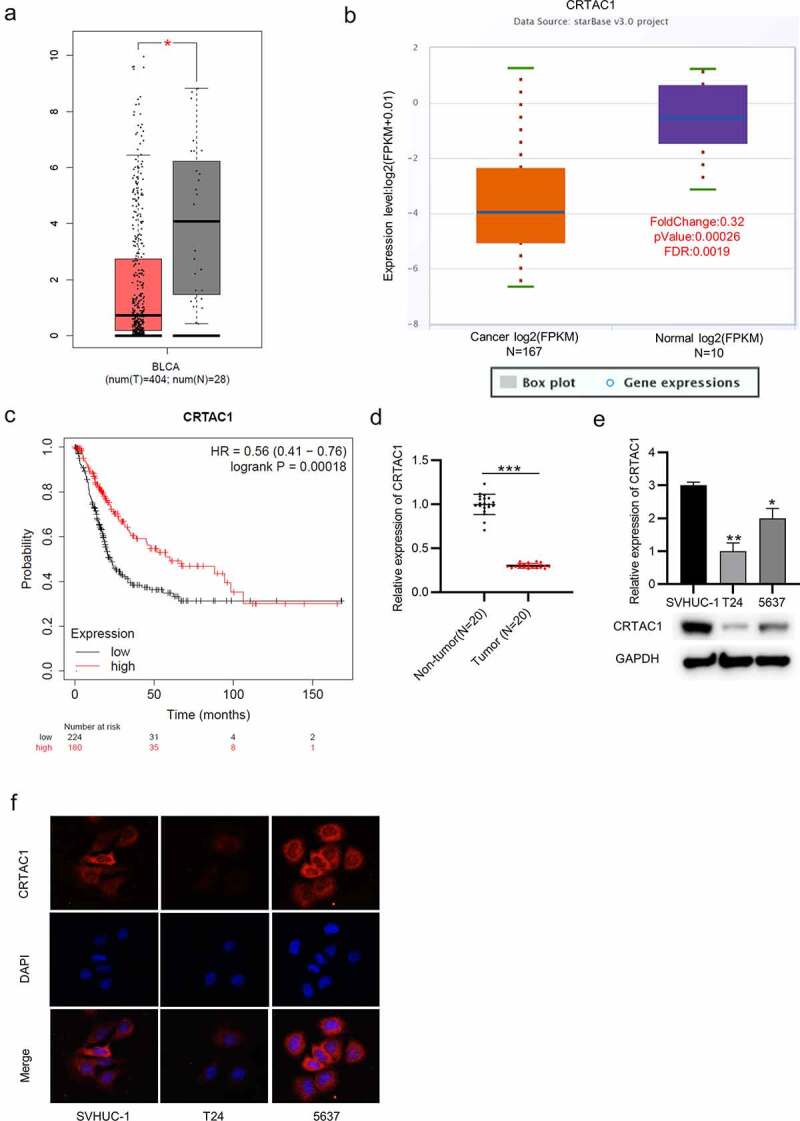
CRTAC1 is downregulated in bladder cancer tissues and cells

The expression pattern of CRTAC1 in bladder cancer was predicted using Gene Expression Profiling Interactive Analysis (GEPIA) (http://gepia.cancer-pku.cn/) and starBase (http://starbase.sysu.edu.cn/) websites. The result indicated that CRTAC1 was significantly downregulated in bladder cancer tissues (Figure 1(a-b)). According to the survival analysis from Kaplan-Meier Plotter, high CRTAC1 expression are associated with better prognosis in patients with bladder cancer (Figure 1(c)). Then, we measured the level of CRTAC1 in 20 pairs of tissue samples collected from patients. The result of RT-qPCR showed that CRTAC1 was lowly expressed in the bladder cancer tissues (Figure 1(d)). Moreover, RT-qPCR and western blotting also revealed the downregulation of CRTAC1 in bladder cancer cell lines compared with that in normal urothelial cell line SVHUC-1 (Figure 1(e)). Immunofluorescence staining assay further demonstrated that CRTAC1 expression was at a low level in T24 and 5637 cells compared with that in normal urothelial cells, and CRTAC1 was mainly located in the cell nucleus (Figure 1(f)).

(a-b) CRTAC1 expression in tissue samples of bladder cancer was predicted by the GEPIA and starBase websites. (c) The survival analysis of bladder cancer patients with high or low expression of CRTAC1 was analyzed by Kaplan–Meier Plotter. (d) CRTAC1 level in bladder cancer tissue samples (n = 20) was detected by RT-qPCR. (e) The mRNA and protein expression of CRTAC1 in bladder cancer cell lines (T24, 5637) and normal urothelial SVHUC-1 cells was examined by RT-qPCR. (F) Immunofluorescence staining was used to assess the expression and location of CRTAC1 expression in bladder cancer cell lines and normal urothelial cell line. *p < 0.05, **p < 0.01, ***p < 0.001.

## CRTAC1 inhibits bladder cancer cell proliferation, migration, invasion and EMT process

**Figure 2.**
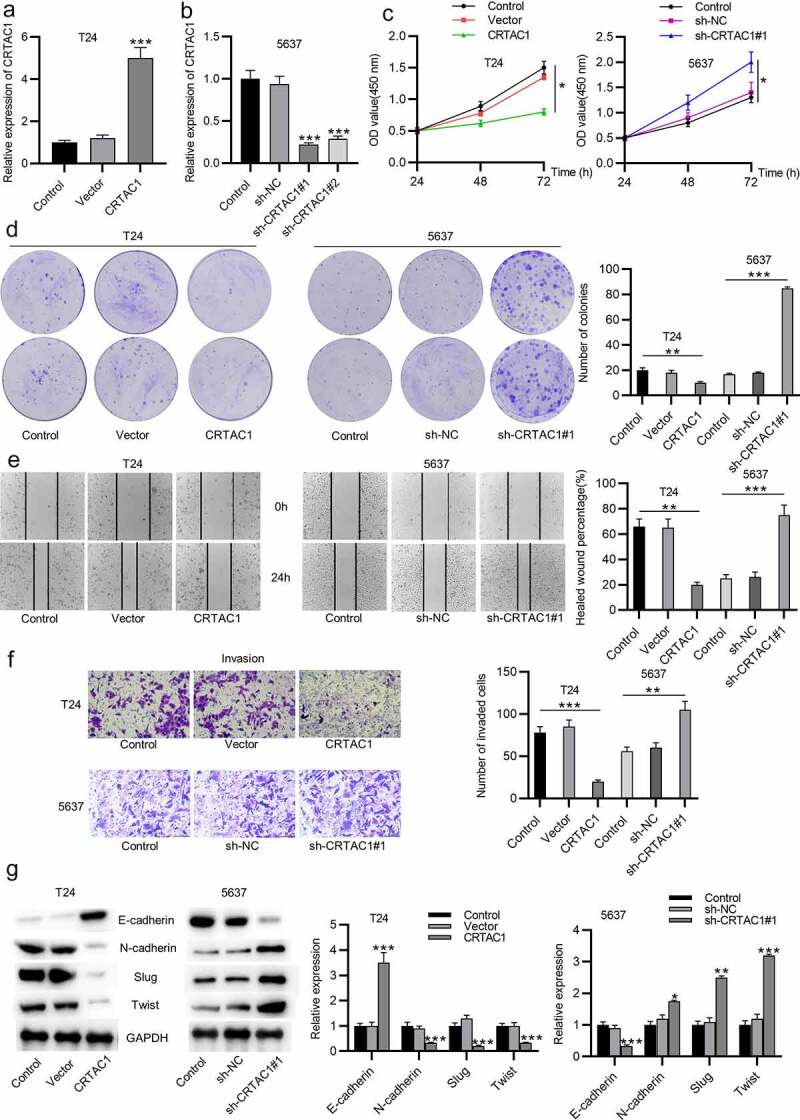
CRTAC1 inhibits bladder cancer cell proliferation, migration, invasion and EMT process

Gain-of-function experiments and loss-of-function experiments were conducted to explore the effect of silenced or overexpressed CRTAC1 on malignant phenotypes of bladder cancer cells. Cell models with high or low level of CRTAC1 were established in bladder cancer cell lines (T24,5637) respectively. The overexpression or knockdown efficiency of CRTAC1 was verified by RT-qPCR in T24 and 5637 cells (Figure 2(a-b)). The results of CCK-8 assays revealed that CRTAC1 overexpression inhibited T24 cell viability, while CRTAC1 depletion enhanced the viability of 5637 cells (Figure 2(c)). According to colony formation assays, the proliferative ability of T24 cells was inhibited by overexpressed CRTAC1, while that of 5637 cells was promoted by CRTAC1 knockdown (Figure 2(d)). Wound healing assays were used for cell migration assessment. The results indicated that the migratory ability of T24 cells was inhibited due to CRTAC1 overexpression, while the migration of 5637 cells was promoted after the transfection of sh-CRTAC1#1/2 (Figure 2(e)). Transwell assays also demonstrated that overexpressed CRTAC1 inhibited the invasion of T24 cells, while silenced CRTAC1 facilitated the invasive capacity of 5637 cells (Figure 2(f)). Then, we measured protein levels of EMT markers in T24 and 5637 cells using western blotting. The protein expression of E-cadherin was elevated and that of N-cadherin, Slug and Twist was reduced by CRTAC1 overexpression in T24 cells, while the levels of these proteins showed opposite changes in 5637 cells after CRTAC1 knockdown (Figure 2(g)).

(a-b) RT-qPCR was used to assess the overexpression or knockdown efficiency of CRTAC1 in T24 and 5637 cells respectively. (c) The viability of T24 and 5637 cells after CRTAC1 overexpression or knockdown was detected using CCK-8 assays. (d) The proliferative ability of T24 and 5637 cells after indicated transfection was detected using colony formation assays. (e) Wound healing assays were conducted to assess the migratory ability of T24 and 5637 cells after CRTAC1 overexpression or knockdown. (f) Transwell assays were performed to detect the invasion of T24 and 5637 cells overexpressing or silencing CRTAC1 expression. (g) Protein levels of EMT markers (E-cadherin, N-cadherin, Slug and Twist) were detected by western blotting in T24 and 5637 cells after the above transfection. *p < 0.05, **p < 0.01, ***p < 0.001.

## CRTAC1 targets YY1 in bladder cancer cells

**Figure 3.**
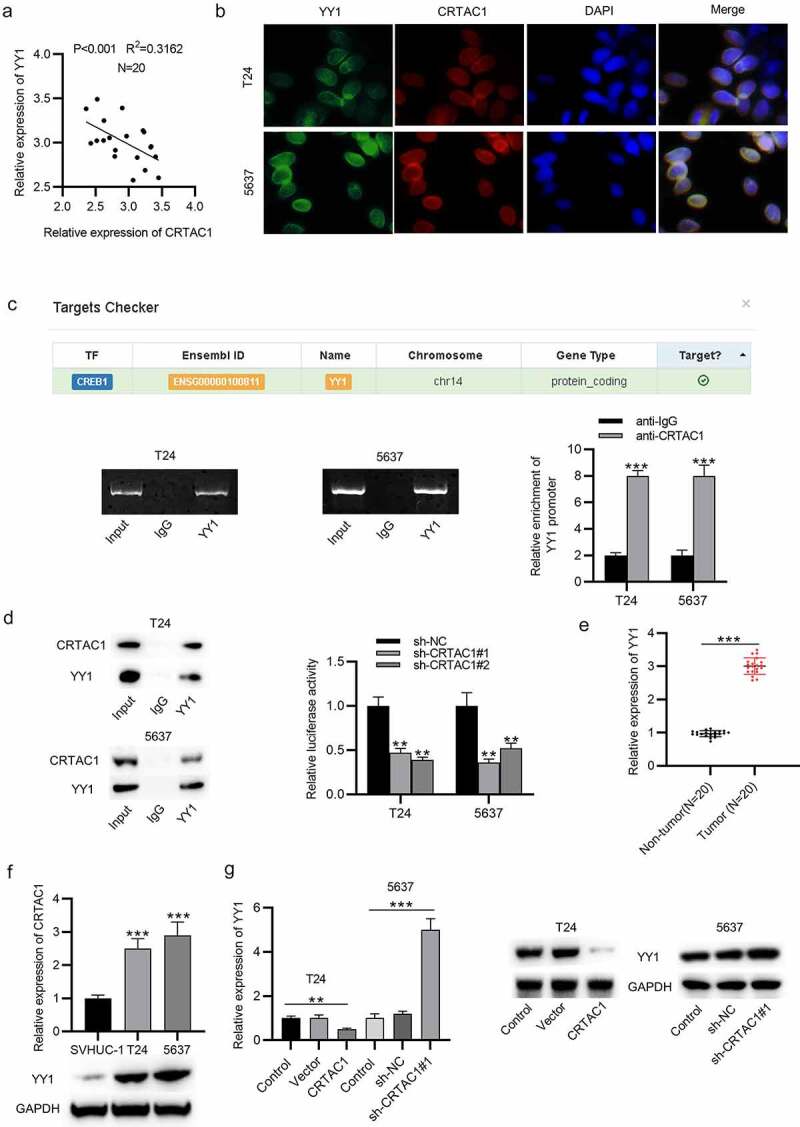
CRTAC1 targets YY1 in bladder cancer cells

Subsequently, we investigated the downstream regulatory mechanism of CRTAC1 in bladder cancer. First, the result of Pearson correlation analysis indicated that the expression of CRTAC1 was negatively associated with YY1 in the tissues of bladder cancer patients (Figure 3(a)). Then, the expression and colocalization of CRTAC1 and YY1 in the nucleus of bladder cancer cells was visualized by immunofluorescent staining assay. The interaction between CRTAC1 and YY1 was identified by ChIP and coimmunoprecipitation (Co-IP) assays (Figure 3(b-c)). Luciferase reporter assays showed that silenced CRTAC1 elevated the luciferase activity of YY1 promoter in T24 and 5637 cells (Figure 3(d)). Then, we measured the expression of YY1 in bladder cancer tissue samples. The result indicated that YY1 was highly expressed in bladder cancer tissues (Figure 3(e)). The mRNA and protein expression of YY1 in bladder cancer cell lines (T24, 5637) was also demonstrated to be higher than that in normal urothelial cell line SVHUC-1 (Figure 3(f)). Moreover, we explored whether CRTAC1 regulated YY1 level in bladder cancer cells. CRTAC1 overexpression decreased the mRNA and protein levels of YY1, while CRTAC1 deficiency showed opposite effects on YY1 expression, which indicated that CRTAC1 negatively regulated YY1 expression in bladder cancer cells (Figure 3(g)).

(a) Pearson correlation analysis was used to assess the expression correlation between CRTAC1 and YY1 in bladder cancer tissues. (b) The immunofluorescent staining was used to detect the expression and colocalization of CRTAC1 and YY1 in the nucleus of bladder cancer cells. (c) The interaction between CRTAC1 and YY1 was explored by ChIP and Co-IP assays. (d) Luciferase reporter assay was used to explore the binding between CRTAC1 and YY1 promoter. (e) YY1 expression in bladder cancer tissue samples was examined by RT-qPCR. (f) The mRNA and protein levels of YY1 in T24, 5637 and SVHUC-1 cells were examined by RT-qPCR and western blotting. (g) YY1 expression in bladder cancer cells after CRTAC1 overexpression or silencing. **p < 0.01, ***p < 0.001.

## CRTAC1 inactivates the TGF-β signaling pathway by downregulating YY1

**Figure 4.**
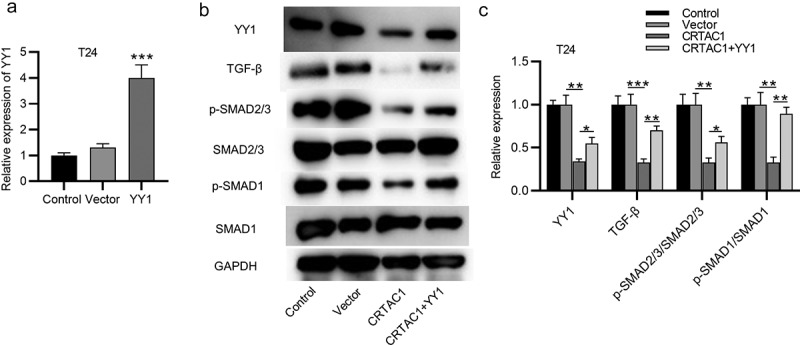
CRTAC1 inactivates the TGF-β signaling pathway by downregulating YY1

YY1 has been reported to activate the TGF-β signaling in bladder cancer and mediate the EMT pathway [16]. We then explored whether CRTAC1 regulated the TGF-β signaling in bladder cancer cells. First, the overexpression efficiency of YY1 in T24 cells was confirmed by RT-qPCR (Figure 4(a)). According to western blotting, the protein expression of TGF-β, p-SMAD2/3 and p-SMAD1 was all decreased by CRTAC1 overexpression, while YY1 overexpression was revealed to reverse the inhibitory effect of overexpressed CRTAC1 on the protein level of p-SMAD2/3/ SMAD2/3 and p-SMAD1/ SMAD1 (Figure 4(b-c)).

(a) The overexpression efficiency of YY1 in T24 cells was detected using RT-qPCR. (b-c) The protein expression of TGF-β, p-SMAD2/3, SMAD2/3, p-SMAD1 and SMAD1 in T24 cells after CRTAC1 overexpression and YY1 overexpression was detected by western blotting. *p < 0.05, **p < 0.01, ***p < 0.001.

## CRTAC1 inhibits proliferation, migration, invasion and EMT of bladder cancer cells by downregulating YY1

**Figure 5.**
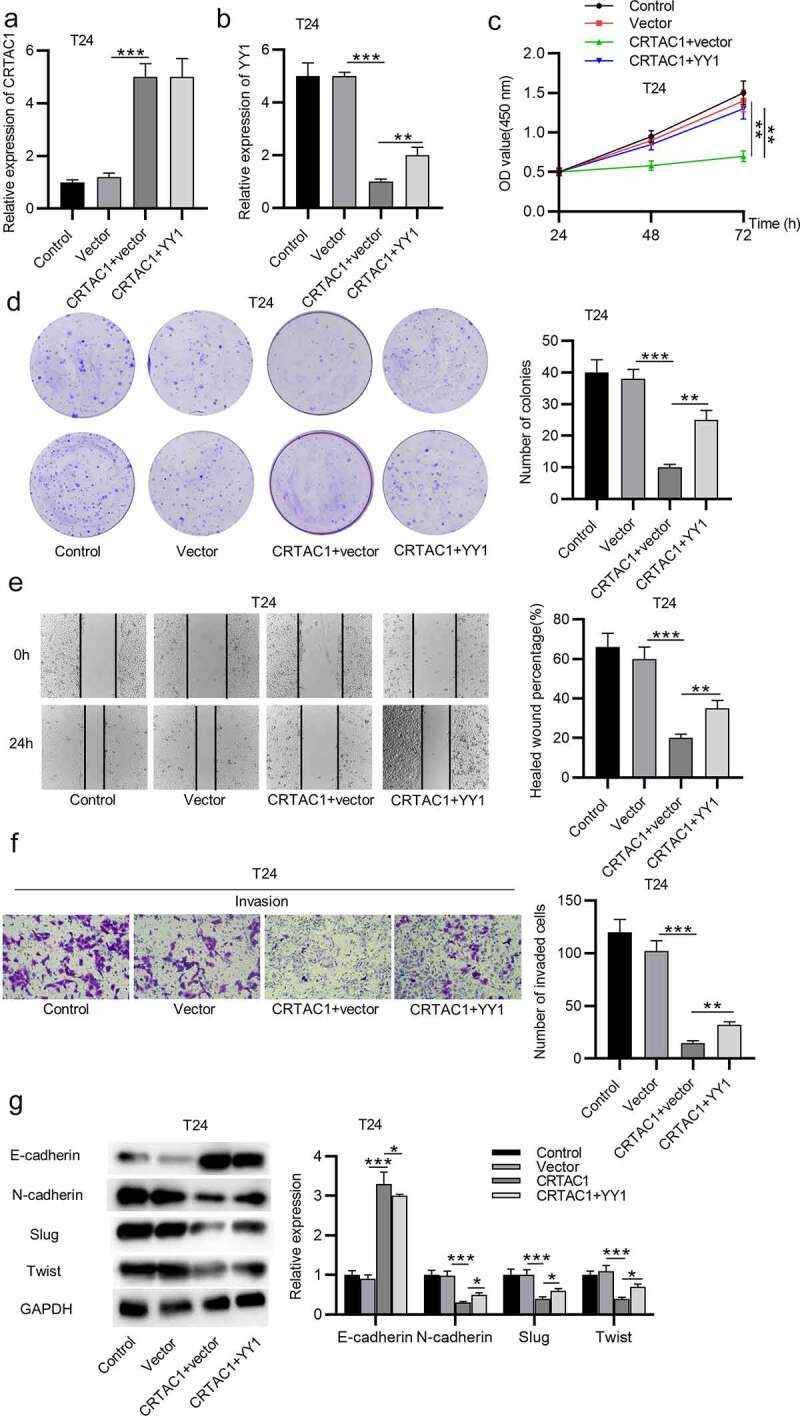
CRTAC1 inhibits malignant behaviors of bladder cancer cells by downregulating YY1

Rescue assays were performed to explore the effect of YY1 on malignant behaviors of bladder cancer cells after CRTAC1 overexpression. The expression of CRTAC1 was elevated by transfection of pcDNA3.1/CRTAC1 in T24 cells, while YY1 expression was decreased after CRTAC1 overexpression (Figure 5(a-b)). After transfecting the overexpression vectors for YY1, the expression of CRTAC1 showed no significant change, while YY1 expression was elevated in T24 cells (Figure 5(a-b)). YY1 overexpression reversed the suppressive effect of overexpressed CRTAC1 on T24 cell viability and proliferation (Figure 5c-d). According to wound healing assays and Transwell assays, CRTAC1 overexpression inhibited the migratory and invasive abilities of T24 cells, while YY1 overexpression offset the inhibitory effect of overexpressed CRTAC1 on cell migration and invasion (Figure 5(e-f)). Western blotting showed that YY1 overexpression reversed the increase in the protein level of E-cadherin and rescued the decrease in protein levels of N-cadherin, Slug and Twist induced by CRTAC1 overexpression in T24 cells (Figure 5(g)).

(a-b) The expression levels of CRTAC1 and YY1 in T24 cells after transfection of pcDNA3.1/CRTAC1, empty pcDNA3.1 vector or co-transfection of pcDNA3.1/CRTAC1 and pcDNA3.1/YY1 were examined by RT-qPCR. (c-d) CCK-8 and colony formation assays were performed to measure the viability and proliferation of T24 cells after the above transfection. (e-f) The migration and invasion of T24 cells after CRTAC1 and YY1 overexpression was assessed by wound healing and Transwell assays. (g) The protein levels EMT markers in T24 cells after the above transfection were detected by western blotting. *p < 0.05, **p < 0.01, ***p < 0.001.

## Discussion

In the current study, we found that the expression of CRTAC1 was decreased in bladder cancer tissue samples and cell lines. High CRTAC1 expression are associated with better prognosis in patients with bladder cancer. Then, the function of CRTAC1 in bladder cancer cells *in vitro* was explored by a series of gain/loss-of-function assays. The results indicated that CRTAC1 overexpression inhibited cell viability, proliferation, migration, invasion and EMT process, while CRTAC1 knockdown showed the opposite effects.

YY1 has been revealed to be highly expressed in various cancers, including prostate cancer [27], ovarian cancer [28], breast cancer [29], osteosarcoma [30], and melanoma [31]. The interaction between CRTAC1 and YY1 was demonstrated in our study. The expression of CRTAC1 and YY1 was negatively correlated with each other in the tissue samples of bladder cancer. According to Immunofluorescent staining and Co-IP assays, colocalization of CRTAC1 and YY1 was identified in the nucleus of bladder cancer cells. The interaction between CRTAC1 and YY1 was further verified using ChIP assay. Moreover, YY1 was expressed at a high level in bladder cancer tissues. The mRNA and protein levels of YY1 were upregulated in bladder cancer cells. Additionally, mRNA and protein expression of YY1 was decreased by CRTAC1 overexpression and increased by CRTAC1 knockdown, which indicated that CRTAC1 negatively regulated YY1 expression in bladder cancer cells.

Furthermore, YY1 has been reported to activate the TGF-β pathway which is a versatile pathway regulating cellular processes such as proliferation, differentiation, and apoptosis [32–35]. SMAD2/3 is recruited to TGF-β1 factor for phosphorylation to activate the transcription of downstream genes. YY1 is reported to promote the TGF-β-induced EMT and pro-fibrosis in pulmonary fibrosis [36]. TGF-β pathway is also indicated to play important roles in bladder cancer. For example, LINC01451 promotes bladder cancer cell proliferation, invasion and EMT by activating the TGF-β/Smad pathway [37]. Nucleolar and spindle associated protein 1 (NUSAP1) facilitates proliferation, migration, invasion and EMT of bladder cancer cells via the activation of the TGF-β signaling [38]. GP73 promotes cell invasion and EMT process via the TGF-β signaling in bladder cancer [39]. In the present study, we demonstrated that YY1 activated the TGF-β signaling while CRTAC1 inhibited the activation of TGF-β signaling by targeting YY1 in bladder cancer cells. YY1 overexpression was revealed to reverse the inhibitory effect of upregulated CRTAC1 on TGF-β signaling and bladder cancer cell viability, proliferation, migration, invasion and EMT process.

In conclusion, CRTAC1 inhibits bladder cancer cell proliferation, migration, invasion and EMT by targeting YY1 to inactivate the TGF-β signaling pathway. The findings of our study may provide clues for the exploration the therapeutic targets of bladder cancer.

However, there are some limitations in our study. First, the upstream regulatory mechanism of CRTAC1 was not explored in bladder cancer cells. Second, the function of CRTAC1 on bladder cancer progression *in vivo* needed further investigation.

## Conclusion

CRTAC1 is downregulated in bladder cancer tissues and cells. CRTAC1 overexpression inhibits cell proliferation, migration, invasion and EMT process, and CRTAC1 deficiency promotes these malignant behaviors of bladder cancer cells. CRTAC1 directly targets YY1 in bladder cancer cells. Moreover, CRTAC1 inactivates TGF-β signaling by downregulating YY1. YY1 overexpression reverses the suppressive effect of CRTAC1 overexpression on malignant behaviors of bladder cancer cells. Overall, CRTAC1 inhibits cell proliferation, migration, invasion and EMT process in bladder cancer by downregulating YY1 to inactivate the TGF-β pathway.

## Data Availability

Data sharing not applicable to this article as no datasets were generated or analysed during the current study.
